# SPREAD OF QUALITY IMPROVEMENT PROJECTS IMPLEMENTED IN A GERIATRICS WORKFORCE DEVELOPMENT PROGRAM

**DOI:** 10.1093/geroni/igz038.2817

**Published:** 2019-11-08

**Authors:** Claire E OHanlon, Claire E O’Hanlon, Debra Saliba, Joseph Douglas, Michael N Mitchell, Carol Callaway-Lane, Josea Kramer

**Affiliations:** 1 VA Greater Los Angeles Healthcare System, Los Angeles, California, United States; 2 Veterans Affairs Center for the Study of Healthcare Innovation, Implementation & Policy, Los Angeles, California, United States; 3 Veterans Affairs Center for the Study of Healthcare Innovation, Implementation & Policy University of California; Los Angeles Borun Center for Gerontological Research, Los Angeles, California, United States; 4 Geriatric Research Education Clinical Center, VA Greater Los Angeles Healthcare System (contractor), North Hills, California, United States; 5 Veterans Affairs Center for the Study of Healthcare Innovation, Implementation & Policy, North Hills, California, United States; 6 Veterans Affairs Tennessee Valley Healthcare System, Geriatric Research Education Clinical Center, Nashville, Tennessee, United States; 7 Veterans Affairs Greater Los Angeles Healthcare System Geriatric Research Education Clinical Center Division of Geriatric Medicine, David Geffen School of Medicine at UCLA, North Hills, California, United States

## Abstract

This project characterizes spread of quality improvement (QI) projects initiated in the U.S. Department of Veterans Affairs (VA) Geriatric Scholars Program (GSP) workforce development program. This mixed methods study analyzes a recent cross-sectional survey of GSP participants and program-level data on participant characteristics and QI project topics. We surveyed 578 scholars who had completed all program requirements to that point, and still worked for VA; 207 (35%) responded. The majority of respondents who had been in the program for at least six months (70%) reported sustainment of their QI project beyond initial implementation and nearly a third (30.4%) reported any spread beyond their own care team. QI project topics spanned many domains and percent of projects reporting spread varied across domains from 0% to 67%. A workforce development capstone activity in which participants demonstrate substantive and methodological competency can foster bottom-up QI activities in a large, diverse health care system.

